# A Lexical Representational Mechanism Underlying Verbal Satiation: An Empirical Study With Rarely Used Chinese Characters

**DOI:** 10.3389/fpsyg.2019.02236

**Published:** 2019-10-02

**Authors:** Kang Cao, Jie Li, Baizhou Wu, Hong Zhang, Hu He

**Affiliations:** ^1^School of Psychology, Inner Mongolia Normal University, Hohhot, China; ^2^School of Music and Education, Chizhou University, Chizhou, China; ^3^Inner Mongolia Autonomous Region Key Laboratory of Psychology, Hohhot, China

**Keywords:** verbal satiation, semantic satiation, lexical satiation, associative satiation, orthographic satiation, repetitive semantic processing

## Abstract

High repetitions of a character induce a feeling of uncertainty of the character. This phenomenon is named as *Verbal Satiation*. However, the locus and nature of the verbal satiation remain controversial. To investigate whether verbal satiation occurs at the lexical representational locus, we used rarely used Chinese characters as stimuli to exclude confounding factor of meaning access. Participants were asked to judge whether or not a single Chinese character such as “
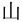
” is a composition of a rarely used Chinese character such as “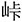.” The experiment consists of 4 sets with 11 blocks in each set. Every 20 trials consist of a block, and the same rarely used characters were repeated in half of these trials. To observe the satiation effect that is offset by practice effect with more statistical power, we did ANOVA analysis for each set. The statistical results revealed that subjects responded differently at different time periods. In the first set, participants responded faster in later trials; After that, reversely, participants responded slower in later trials; Then they responded slower for the repeated characters in middle trials; Finally, participants responded slower for the repeated characters without regard to the trial position. These results show a competition process between satiation effect and practice effect and reveal that the verbal satiation can occur at a lexical representational locus.

## Introduction

In daily life, following massed overt repetition of a character, we usually have a peculiar sense of uncertainty of the meaning or a peculiar sense of unfamiliarity with the orthographic composition of the character (Kuhl and Anderson, 2011; Cheng and Lin, 2013). This phenomenon is known as *verbal satiation* (Lambert and Jakobovits, 1960), and was first reported by Severance and Washburn (1907), who showed that participants would report their loss of meaning after fixating at a word for 3 min.

The Responding Optimally with Unknown Sources of Evidence (ROUSE) theory may provide an explanation for the general cognitive mechanism under the satiation effect. Imagine that we are reading a sentence, the information retrieved automatically from visually presented characters can last for a period of time, so, the information from previous characters and the information from present characters are mixed together. It is difficult to distinguish the source of information between previous and present characters, which means that a source confusion happened. ROUSE theory proposed that the source confusion can be reduced by discounting the information from previous characters (Huber et al., 2002). This discounting mechanism of information produces a deficit in processing repetitions (Tian and Huber, 2010), meaning that the satiation effect occurs.

We are focusing on the important questions concerning the locus and nature of verbal satiation produced by the discounting mechanism of information. There are three divergent theoretical descriptions that answer these questions: (1) The term *semantic satiation* emphasizes the locus of verbal satiation is thought to be semantic (Lambert and Jakobovits, 1960; Smith and Klein, 1990; Pynte, 1991; Black, 2001); (2) The term *lexical satiation* makes a point that the “loss of meaning” is dependent upon the changes in the processing of the orthographic or the phonological representation of the word (Esposito and Pelton, 1971; Tian and Huber, 2010); (3) *Associative satiation* states that the less efficient association between the lexical representation entry and its associated meaning results in the verbal satiation (Tian and Huber, 2010; Yuan et al., 2017).

Earlier, the debate mainly took place between semantic satiation and lexical satiation. The early studies that relied upon self-report or introspection showed a loss of meaning (Bassett et al., 1919) and changes in semantic ratings (Lambert and Jakobovits, 1960) after repeating the same word aloud. To exclude report bias, Smith and Klein (1990) used reaction time as dependent measures. They found that subjects responded slower after 30 repetitions of the repeated category, and concluded semantic satiation. On the other side of arguments, Pilotti et al. (1997) concluded a suggestive (Kounios et al., 2000) point that the verbal satiation was induced by auditory characteristic perceptual changes. For the non-alphabetic characters, such as Chinese characters, the rate of verbal satiation was sensitive to the orthographic structures, and just as the term *orthographic satiation* suggests, the feeling of uncertainty about characters occurs at the locus of lexical orthographic representation (Cheng and Lin, 2013).

All the experiments mentioned above used two separate tasks, one to produce satiation and one to measure satiation. This method may introduce task-switching effects, and pollute the research results (Tian and Huber, 2010). By using a single task that both induces and measures satiation effect, Tian and Huber (2010) conducted a series of experiments to test at which locus satiation effect occurs. The result of their first experiment supports both lexical and semantic accounts of verbal satiation, but the second and the third support neither of them, hence, they concluded associative satiation, which means that the verbal satiation is on account of association between lexical entry and associated meaning. By applying the same experimental paradigm to Chinese characters, researchers deducted the same conclusion (Yuan et al., 2017).

However, previous studies seem to have overlooked the fact that the meaning access and practice effect influence the research results, and lead to the satiation effect be obscured. For one thing, because the subjects know the meaning of characters, the influence of meaning access cannot be entirely excluded. Participants can complete the word matching task based on top-down processing, meaning that participants completed this task based on their knowledge or concept, but not based on the lexical representations of the characters. The top-down processing can facilitate reaction (Bar, 2003), so, it is possible that the satiation effect is offset by it. To make matters worse, even if the satiation is found in these researches, we cannot distinguish that based on the lexical representations from that based on the knowledge or concept. Using rarely used Chinese characters as experimental materials provide a chance to check if lexical satiation exists with two advantages: (1) Participants do not know the meaning of the character, and consequently exclude the influence of the meaning access. (2) Chinese characters can be decomposed into different radicals, for example, “
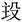
” can be decomposed into three radicals “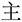,” “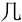,” and “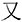.” If we ask participants to complete the cue-target matching task by using “
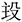
” as cue character and “
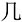
” as target character, the participants’ task processing can be confined to the lexical level, and the satiation must be lexical satiation. For another, due to the repeated task, the practice effect accelerates the reaction, but the satiation effect decelerates it. It is possible that the satiation effect is offset by practice effect over the course of time. This “offset effect” leads to insufficient statistical power to observe the satiation effect, therefore, it is inadequate to infer that there was no lexical satiation due to non-significant statistical results. We cannot exclude the practice effect by the experimental design, but, fortunately, the features that the practice effect has its upper limit and satiation effect can be accumulated during the time (Tian and Huber, 2010; Cheng and Lan, 2011; Yuan et al., 2017) provide a chance to counter it by improving the statistical analysis procedure.

Briefly, in the present study, we conducted an experiment to directly test whether or not verbal satiation occurs at the lexical locus with two improvements: First, by using the rarely used Chinese characters, the present experimental design can exclude the impact of meaning access and confine participants’ task processing to the orthographic level. Second, we also improved the statistical procedures to provide a relatively sufficient power to observe the lexical satiation effect.

Chinese characters are ideograms that have similarities and differences in cognitive processing compared with phonetic characters (Chee et al., 1999, 2000). Most of the studies about verbal satiation are based on phonograms such as English characters, and there are few studies based on ideograms such as Chinese characters. Based on these few researches about verbal satiation on Chinese characters, we predict that the lexical satiation does exist in the present experiment, and it is valuable for understanding the nature of verbal satiation.

## Materials and Methods

### Participants

A total of 18 undergraduate/graduate students (6 males; mean age, 20.89 years; range, 18–24) were recruited for this study. All were native Mandarin Chinese speakers with normal or corrected-normal vision. They all received ¥20 in exchange for their participation. This study was carried out in accordance with the recommendations of the operating guidelines of the School of Psychology Ethics Committee (Inner Mongolia Normal University). All subjects gave written informed consent in accordance with the Declaration of Helsinki. The protocol was approved by the School of Psychology Ethics Committee (Inner Mongolia Normal University).

### Stimuli

Eleven rarely used Simplified Chinese characters, such as “
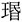
” (fine jade), “
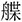
” (boat), were selected. All the rarely used characters belong to left-right structure, and each consist of three commonly used characters (e.g., “
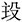
” is composed of “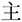,” “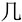,” and “
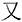
”; “
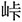
” is composed of “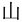,” “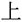,” and “
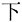
”).

Participants were asked to judge if a commonly used character was a composition of the corresponding rarely used character. In a matching trial, a commonly used character is a composition of a rarely used character, such as “
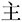
” is a composition of “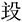.” Accordingly, we assigned the selected three commonly used mismatching characters for each rarely used character (e.g., “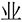,” “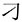,” “
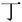
” are three assigned mismatching characters for “
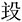
”), and each has the same number of strokes as a corresponding matching character (e.g., for “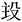,” mismatching character “
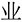
” also has five strokes as its matching character “
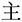
”). All the rarely used characters and their corresponding matching and mismatching characters are listed in Supplementary Appendix A1.

### Procedure

**FIGURE 1:**
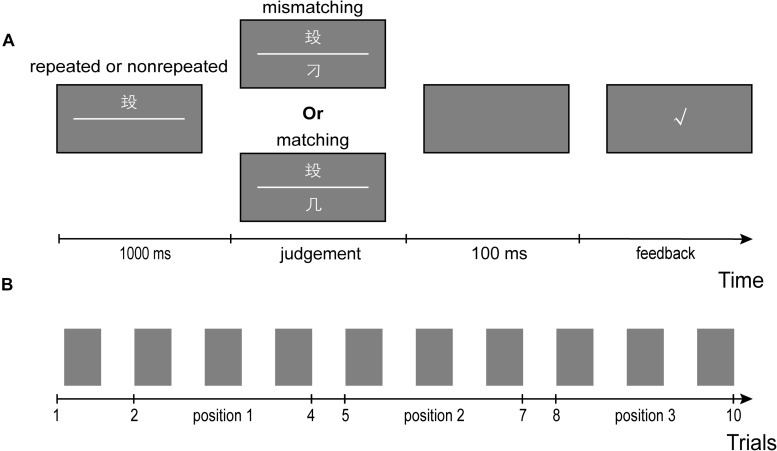
Experimental paradigm. **(A)** An illustration of the stimuli and procedure of a trial. In a block, which consists of randomly arranged 20 trials, a rarely used character was selected to be presented as cue character in 10 trials repeatedly, and others were selected to be presented as cue character once in the rest 10 trials. In either repeated or non-repeated 10 trials, 5 target characters are matching and other 5 target characters are mismatching. **(B)** An illustration of trial positions in a sequence of repeated or non-repeated 10 trials. From the second trial to the tenth trial, every 3 trials indicate a trial position.

The experimental paradigm showed in Figure 1 is similar to that reported by Tian and Huber (2010). The participants sat comfortably about 60 cm in front of a laptop monitor. In each trial, a rarely used character was presented above the midline of the screen for 1000 ms. Then, a corresponding matching or mismatching character was presented below the midline while the rarely used character remained on the screen. These two characters were always presented until participants responded. Participants were asked to judge if the character below was a composition of the character above as quickly and accurately as possible. The “F” and “J” key on an external USB keyboard indicated a matching or mismatching response. The mapping keys were counterbalanced across participants. After the participants’ response, the screen went blank for 100 ms, and then a green check or red cross feedback was presented randomly for 500–1000 ms before the next trial began.

There were 20 trials per block. In each block, one of the 11 rarely used characters were randomly chosen to repeat in 10 trials as cue characters making the repeated condition, and the rest 10 rarely used characters were used as cue characters in the other 10 trials producing the non-repeated condition. The order of these 20 trials is random. In either repeated or non-repeated condition, half of the trials were matching trials, and the other half of trials were mismatching trials. In the repeated condition, we only have 3 matching or mismatching commonly used characters for each rarely used character, so, the five matching or mismatching commonly used characters were selected according to the following two steps: (1) replicate the 3 matching or mismatching commonly used characters twice, and then we had 6 commonly used characters; (2) exclude one from the 6 commonly used characters randomly. In the non-repeated condition, the matching or mismatching character was selected randomly from the three commonly used characters each time.

The experiment started with 20 practice trials. A participant whose accuracy is less than 90% would practice 20 trials again until the accuracy reaches 90%. The stimuli used in the practice stage were not used in the following experimental trials.

All participants completed 4 sets with 11 blocks in each set, and each rarely used character served as the dominant repeated stimuli once for each block. The presentation sequence of blocks in each set and the pairing of rarely used and commonly used characters in each block were randomized. There was a compulsory rest of at least 90 s after every set. All participants reported that they did not know the meaning of the rarely used characters after the experiment.

## Results

The core statistical analysis procedures are similar to those reported by Tian and Huber (2010). No participant in the present study was excluded due to overall accuracy under 90%. For each participant’s data, reaction times that less than 300 ms and greater than 1500 ms were excluded, and only correct reactions were analyzed. In the sequence of 10 trials in the repeated or non-repeated condition, the first trial was not analyzed. The later nine trials were broken into 3 segments (2–4, 5–7, and 8–10). As a result, the experimental design was a 2 × 2 × 3 repeated-measures factorial design, and the three factors were repetition status (repeated vs. non-repeated), matching status (matching vs. mismatching), and position (Trials 2–4, 5–7, and 8–10 in a sequence).

**FIGURE 2:**
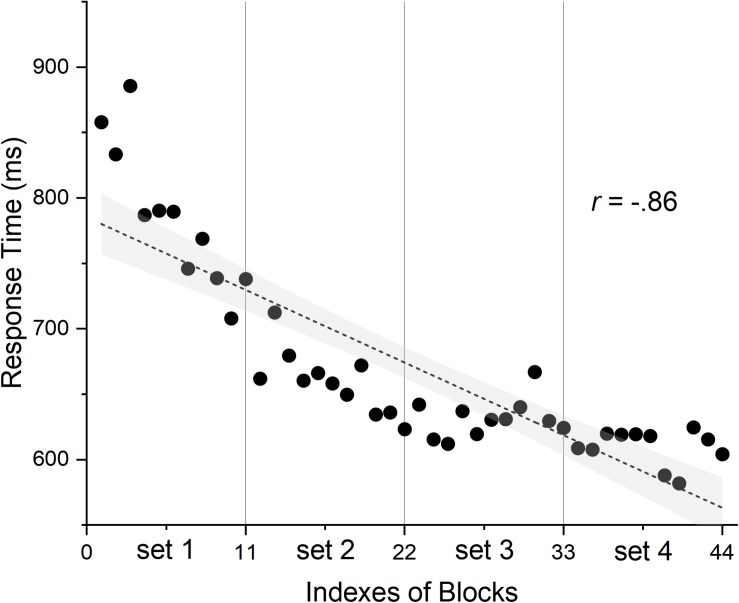
The relationship between indexes of blocks and response time. Shadow area represents 95% confidence bands; 4 sets consist of every 11 blocks divided by the vertical lines.

In the analysis procedures mentioned above, the practice effect which can offset the satiation effect may be neglected. To verify if there was a practice effect, we drew a Pearson correlation analysis between the index of 44 blocks and the mean of median response time (RT) for each block across participants. As shown in Figure 2, the result, *r* = −0.86, *p* < 0.001, showed that the participants’ responses were getting faster and faster over the course of time and revealed a practice effect.

However, participants’ response would be slowed down due to the satiation effect, and be facilitated for the practice effect over the course of time. Therefore, a competing process between practice effect and satiation effect occurs, and the statistical analysis should not ignore the variable of the time course. We used the indexes of 4 sets as a factor to indicate the time course in the following statistical analysis procedures for two reasons: (1) Except for the presentation sequence of blocks and the paring of rarely used and commonly used characters in each block, all the 220 trials in one set was a replica of other sets; (2) As an independent variable with 4 levels, it was suitable for the statistical analysis. Consequently, the experimental design became a 4 × 2 × 2 × 3 repeated-measures factorial design and the four variables were set (Set 1–4), repetition status, matching status and position.

We averaged the median RTs across matching status to increase reliability under the premise that there was no interaction effect between matching status and any other variables (all *p*s were greater than 0.05, see Table 1; Tian and Huber, 2010), then we drew a three-way repeated measures ANOVA with set, repetition status, and position.

**TABLE 1:** The results of the interaction effect between matching status and other factors.

**Interaction Effect**	***F***	***p***	**$\eta_{\text{p}}^{2}$**
Set × Matching status	*F*(3, 51) = 2.10	0.11	0.11
Position × Matching status	*F*(2, 34) = 0.11	0.90	0.01
Repetition status × Matching status	*F*(1, 17) = 0.07	0.79	0.00
Set × Position × Matching status	*F*(6, 102) = 1.33	0.25	0.07
Set × Repetition status × Matching status	*F*(3, 51) = 0.76	0.52	0.04
Position × Repetition status	*F*(2, 34) = 1.33	0.28	0.07
× Matching status			
Position × Repetition status × Set	*F*(3.24, 55.10) = 0.19	0.92	0.01
× Matching status^∗^			

*^∗^Mauchly’s test indicated that the assumption of sphericity had been violated, χ^2^(20) = 43.33, *p* = 0.002. Therefore, degrees of freedom were corrected using Greenhouse–Geisser estimates of sphericity (ε = 0.54).*

As mentioned above, there may be a competing process between practice effect and satiation effect, so we predicted that set would interact with repetition status and position, which means that the satiation effect behaved differently over the course of time. The significant Set × Repetition Status × Position interaction confirmed this prediction, *F*(6,102) = 2.53, *p* = 0.03, $\eta_{\text{p}}^{2}$ = 0.13.

The focused result was a two-way interaction between repetition status and position which could indicate the satiation effect for the repeated condition as a function of the number of repetitions of the rarely used character. To observe the verbal satiation effect with sufficient power, we conducted a two-way repeated measures ANOVA with repetition status and position for each set under the premise that set interacted with repetition status and trial position. We performed the same ANOVA with accuracy to test if the change seen with response time was due to speed-accuracy tradeoff. Tables 2, 3 show the median RT and accuracy results for all 12 conditions in each set.

**TABLE 2:** Response time (ms) and standard error of the mean (SE) for all conditions in the 4 sets.

**Sets**	**Conditions**	**Trial 2–4**		**Trial 5–7**		**Trial 8–10**	
		***M***	***SE***	***M***	***SE***	***M***	***SE***
1	Repeated matching	772	27	745	31	762	29
	Repeated mismatching	815	35	779	30	800	40
	Non-repeated matching	761	31	771	41	736	31
	Non-repeated mismatching	833	38	795	32	768	32
2	Repeated matching	657	25	670	22	675	29
	Repeated mismatching	670	22	688	26	709	28
	Non-repeated matching	658	27	679	30	689	34
	Non-repeated mismatching	682	33	664	26	694	28
3	Repeated matching	625	21	624	19	617	22
	Repeated mismatching	634	23	667	31	652	29
	Non-repeated matching	631	23	611	18	623	23
	Non-repeated mismatching	656	28	628	21	658	25
4	Repeated matching	603	19	598	23	625	22
	Repeated mismatching	614	21	628	20	634	20
	Non-repeated matching	592	18	583	21	604	22
	Non-repeated mismatching	618	23	622	20	614	22

**TABLE 3:** Accuracy rate and standard error of the mean (SE) for all conditions in the 4 sets (%).

**Sets**	**Conditions**	**Trial 2–4**		**Trial 5–7**		**Trial 8–10**	
		***M***	***SE***	***M***	***SE***	***M***	***SE***
1	Repeated matching	98	1.08	95	1.38	98	0.96
	Repeated mismatching	98	0.80	97	0.87	97	1.24
	Non-repeated matching	94	1.28	96	1.35	93	1.85
	Non-repeated mismatching	99	0.61	97	0.96	98	0.67
2	Repeated matching	97	1.38	96	1.26	97	1.43
	Repeated mismatching	98	1.09	98	0.84	99	0.70
	Non-repeated matching	96	1.11	98	0.79	96	1.40
	Non-repeated mismatching	99	0.62	99	0.61	99	0.60
3	Repeated matching	98	1.15	98	0.89	97	1.10
	Repeated mismatching	99	0.58	100	0.43	99	0.83
	Non-repeated matching	97	1.08	96	1.07	96	1.11
	Non-repeated mismatching	100	0.37	99	0.72	98	0.87
4	Repeated matching	98	1.14	97	1.20	98	0.90
	Repeated mismatching	98	0.75	99	0.57	99	0.50
	Non-repeated matching	98	1.16	97	1.03	97	1.48
	Non-repeated mismatching	99	0.46	99	0.39	98	1.09

**FIGURE 3:**
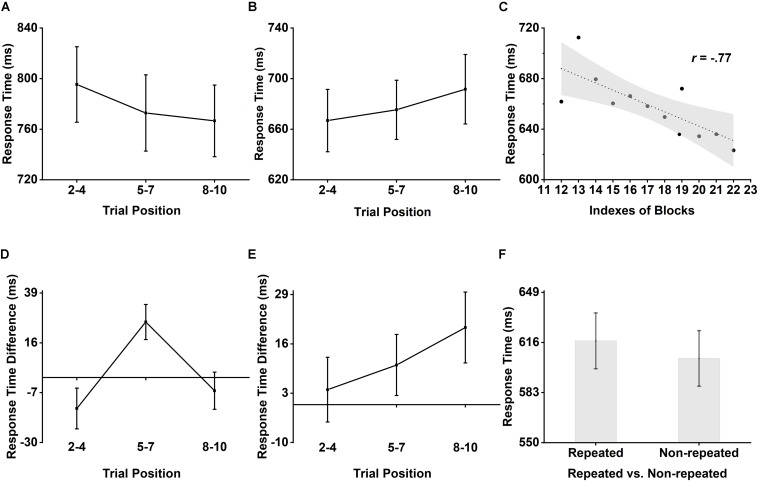
Participants’ behavioral performance in each set. **(A)** Response time result as a function of position in set 1. Bars represent 1 SEM. **(B)** Response time result as a function of position in set 2. Bars represent 1 SEM. **(C)** The relationship between indexes of blocks and response time in set 2. Shadow area represents 95% confidence bands. **(D)** Response time in repeated condition minus response time in non-repeated condition result as a function of position in set 3. Bars represent 1 SEM. **(E)** Response time in repeated condition minus response time in non-repeated condition result as a function of position in set 4. Bars represent 1 SEM. **(F)** Response time in repeated and non-repeated condition. Bars represent 1 SEM.

In the first set, as indicated by the decreasing trend seen in Figure 3A, there was a significant main effect of position, *F*(2,34) = 4.41, *p* = 0.02, $\eta_{\text{p}}^{2}$ = 0.21, and a significant linear trend, *F*(1,17) = 5.96, *p* = 0.03, $\eta_{\text{p}}^{2}$ = 0.26, implying that the responses were getting faster and faster across trial positions. However, the same repeated measures two-way ANOVA on accuracy showed no significant main effect of position, *F*(2,34) = 0.48, *p* = 0.63, $\eta_{\text{p}}^{2}$ = 0.03, indicating that the speed-accuracy tradeoff cannot explain the main effect of position on response time.

In the second set, as indicated by the increasing trend seen in Figure 3B, there was a significant main effect of position, *F*(2,34) = 4.42, *p* = 0.02, $\eta_{\text{p}}^{2}$ = 0.21, and a significant linear trend, *F*(1,17) = 5.88, *p* = 0.03, $\eta_{\text{p}}^{2}$ = 0.26, showing a general response slowing as a function of trial positions. Again, there was no significant main effect of position on accuracy, *F*(2,34) = 0.04, *p* = 0.96, $\eta_{\text{p}}^{2}$ = 0.00, indicating that the general response slowing with increasing position cannot be explained by speed-accuracy tradeoff. The significant relationship between indexes of blocks and response time, Pearson *r* = −0.77, *p* = 0.01, as shown in Figure 3C, indicated that the main effect of position on response time also cannot be explained by fatigue effect.

In the third set, there was a significant interaction effect between repetition status and position as shown in Figure 3D, *F*(2,34) = 5.86, *p* = 0.01, $\eta_{\text{p}}^{2}$ = 0.26, then we tested the simple main effect of repetition status in different trial positions, the result revealed that at the second position (Trial 5–7), participants response was slower in repeated condition than it was in non-repeated condition (645 ms vs. 620 ms), *F*(1,17) = 10.06, *p* = 0.01 (Bonferroni corrected), $\eta_{\text{p}}^{2}$ = 0.37. The same ANOVA on accuracy showed that the participants’ response accuracy was higher in repeated condition than it was in non-repeated condition (98.4% vs. 97.6%), *F*(1,17) = 4.75, *p* = 0.04, $\eta_{\text{p}}^{2}$ = 0.22. However, there was no interaction effect between repetition status and position, *F*(2,34) = 0.88, *p* = 0.43, $\eta_{\text{p}}^{2}$ = 0.05. Therefore, the change of response time followed by the repetition status and the position was not due to speed-accuracy tradeoff.

In the last set, there was only a main effect of repetition status on response time, *F*(1,17) = 5.36, *p* = 0.03, $\eta_{\text{p}}^{2}$ = 0.24, indicating that participants responded more slowly in repeated condition than non-repeated condition (617 ms vs. 605 ms). The results were also shown in Figures 3E,F. The speed-accuracy tradeoff also cannot explain the main effect found in response time due to a non-significant main effect of repetition status on accuracy, *F* (1,17) = 0.02, *p* = 0.90, $\eta_{\text{p}}^{2}$ = 0.00.

## Discussion

The present study determined whether or not the verbal satiation occurs at the lexical locus by using a variant speeded category-matching task with rarely used Chinese characters. The experimental results evoked from improved statistical analysis are consistent with our prediction.

The most striking finding of our work is that the lexical satiation effect does exist under the present experimental conditions, and it changes across the course of time due to the competing process between satiation effect and practice effect. This tug-of-war like competition shows up in the results: (1) The practice effect won the competition in the first set reflected in the decreased response time across trial positions. (2) Starting from the second set, the satiation effect gets the upper hand, i.e., the general slowing response as a function of trial position represent the satiation effect. (3) Both sides became evenly matched in the third set reflected in the complicated interaction result, and the satiation effect reflected in the slower response in repeated condition appeared in the second trial position. (4) The practice effect may reach its upper limit in the last set while the satiation effect accumulates through time. The satiation effect finally takes advantage as reflected in the more slowly response in repeated condition than in non-repeated condition.

These results further support Cheng and Lan’s (2011) view that there is an orthographic satiation in Chinese characters. The experimental design and the statistical analysis program they developed are ingenious. However, they did not take measures to avoid the meaning access, so it is difficult to distinguish their results from orthographic satiation to semantic satiation. Our experimental design, i.e., using rarely used Chinese characters as stimuli remedied this defection. Noted also that, our result was different from previous similar researches, Tian and Huber (2010) and Yuan et al. (2017) argued that the satiation effect is due to a loss of association between the lexical representation and its meaning based on the appearance of the satiation effect in their experiment 1, and the absence of lexical satiation and semantic satiation in experiment 2 and 3. The absence of lexical satiation is inconsistent with the appearance of lexical satiation in the present study. The reason may be the confounding factors mentioned above and insufficient statistical power.

Moreover, the different cognitive strategy participants adopted cannot provide additional interpretation of the present results. First, the rarely used characters can be treated as pictures, and the semantic locus of satiation with pictures (Lewis and Ellis, 2000) may confound the present conclusion. Previous researches have shown that texts and pictures are processed in different ways at the early stage, even though they share the same semantic system (Federmeier and Kutas, 2001; Bright et al., 2004; Shinkareva et al., 2011; Devereux et al., 2013). Extend to the present study, the rarely used characters are meaningless for participants, but they are processed differently with pictures at lexical level (early stage), so, even though texts and pictures share the same semantic system, the result in the present experiment cannot be explained as the semantic locus of satiation with pictures. What’s more, participants may adopt an additional strategy by decomposing the rarely used characters into three radicals, keeping them in mind, and then comparing them to the presented target character. This strategy does not provide an additional explanation for the present results but does make experimental results more reliable. Firstly, the present study measured the satiation effect for rarely used characters but not radicals, so the semantic satiation due to the meaning access of radicals has nothing to do with the present interpretation. Secondly, even if it has an effect on the present result, it is a constant variable, and also does not affect the interpretation of the present results. Finally, this strategy confines participants’ cognitive operations to the lexical level and makes the results more reliable.

Tian and Huber (2010) deducted the associative satiation with an excellent example, they wrote:

“For example, … (e.g., ‘lied’ followed by ‘died’, such as in ‘Because the pharmaceutical company lied about the side effects, many patients died’). Loss of association allows the reader to process ‘died’ as a unique occurrence while still building up meaning across the sentence. This is because the shared perceptual elements (‘-ied’) are discounted by reducing their link to the meaning of the first word (e.g., to withhold the truth) while preserving their ability to link to the second word (e.g., end of life). … If there was direct discounting of perception, then it would be difficult to process the letters of ‘died’. If there was direct discounting of meaning, then the meaning of the first word would be lost before the end of the sentence.”

As mentioned above, the ROUSE theory provides an interpretation for the cognitive mechanism of satiation effect. Because of the absence of meaning, the satiation in the present experiment is only lexical satiation. For the verbal satiation phenomenon, if there is only lexical satiation or semantic satiation, it cannot explain the example they wrote above; If there is only associative satiation, it cannot explain the results in the present experiment. To overcome this contradiction, we argue that discounting of information can occur at any level of the lexical level, the associative level, and the semantic level. Consequently, the locus of verbal satiation can be either lexical satiation, associative satiation or semantic satiation, or any combination of them.

Nevertheless, there are also deficits and areas in need of future research: First, the number of stimuli characters were rather minimal. Due to the difficulty in hunting for the rarely used characters that can be decomposed into three compositions, we used 11 cue characters and 6 target characters rather than 16 cue stimuli and 20 target stimuli in previous researches (Tian and Huber, 2010; Yuan et al., 2017). However, the results drawn from the present study showed that our design still offers enough power to study the satiation effect. Second, the perceptual process includes not only lexical but also phonetic representation. Despite the shortage that we do not examine the satiation effect with the phonetic information of Chinese characters, we still can draw the conclusion that verbal satiation can be dependent upon the changes in the processing of the orthographic representation due to the fact that participants do not know the phonetic information about the rarely used characters. In the end, the phenomenon of Chinese characters satiation is specific to the perception of Chinese characters and different from that of objects, human faces, and alphabetic language such as English words (Cheng and Lan, 2011). Indeed, a large body of evidence also showed that the information processing in logographic languages, such as Chinese, differs from alphabetic languages, such as English (Tan et al., 2001, 2005). Further research is needed to check if the satiation effect in logographic language differs from the alphabetic language.

## Data Availability Statement

The raw data supporting the conclusions of this manuscript will be made available by the authors, without undue reservation, to any qualified researcher.

## Ethics Statement

This study was carried out in accordance with the recommendations of the operating guidelines of the School of Psychology Ethics Committee (Inner Mongolia Normal University) with written informed consent from all subjects. All subjects gave written informed consent in accordance with the Declaration of Helsinki. The protocol was approved by the School of Psychology Ethics Committee.

## Author Contributions

KC and JL designed the study, analyzed the data, and wrote the first draft of the manuscript. BW helped designing the study and organized data acquisition, and discussed the first draft of the manuscript. HZ helped analyzing the data and discussed the first draft of the manuscript. HH discussed the first draft of the manuscript.

## Conflict of Interest

The authors declare that the research was conducted in the absence of any commercial or financial relationships that could be construed as a potential conflict of interest.

## References

[B1] Bar M. (2003). A cortical mechanism for triggering top-down facilitation in visual object recognition. *J. Cogn. Neurosci.* 15 600–609. 10.1162/089892903321662976 12803970

[B2] Bassett M. F. Warne C. J. Titchener E. B. Weld H. P. (1919). Minor studies from the psychological laboratory of cornell university: on the lapse of verbal meaning with repetition. *Am. J. Psychol.* 30 415–418.

[B3] Black S. R. (2001). Semantic satiation and lexical ambiguity resolution. *Am. J. Psychol.* 114 493–510. 10.2307/1423607 11789337

[B4] Bright P. Moss H. Tyler L. K. (2004). Unitary vs multiple semantics: PET studies of word and picture processing. *Brain Lang.* 89 417–432. 10.1016/j.bandl.2004.01.010 15120534

[B5] Chee M. W. L. Caplan D. Soon C. S. Sriram N. Tan E. W. L. Thiel T. (1999). Processing of visually presented sentences in mandarin and english studied with fMRI. *Neuron* 23 127–137. 10.1016/s0896-6273(00)80759-x 10402199

[B6] Chee M. W. L. Weekes B. Lee K. M. Soon C. S. Schreiber A. Hoon J. J. (2000). Overlap and dissociation of semantic processing of chinese characters, english words, and pictures: evidence from fMRI. *NeuroImage* 12 392–403. 10.1006/nimg.2000.0631 10988033

[B7] Cheng C.-M. Lan Y.-H. (2011). An implicit test of chinese orthographic satiation. *Read. Writ.* 24 55–90. 10.1007/s11145-009-9201-y

[B8] Cheng C.-M. Lin S.-Y. (2013). Chinese orthographic decomposition and logographic structure. *Read. Writ.* 26 1111–1131. 10.1007/s11145-012-9408-1

[B9] Devereux B. J. Clarke A. Marouchos A. Tyler L. K. (2013). Representational similarity analysis reveals commonalities and differences in the semantic processing of words and objects. *J. Neurosci.* 33 18906–18916. 10.1523/JNEUROSCI.3809-13.2013 24285896 PMC3852350

[B10] Esposito N. J. Pelton L. H. (1971). Review of the measurement of semantic satiation. *Psychol. Bull.* 75 330–346. 10.1037/h0031001

[B11] Federmeier K. D. Kutas M. (2001). Meaning and modality: influences of context, semantic memory organization, and perceptual predictability on picture processing. *J. Exp. Psychol.* 27 202–224. 10.1037//0278-7393.27.1.202 11204098

[B12] Huber D. E. Shiffrin R. M. Quach R. Lyle K. B. (2002). Mechanisms of source confusion and discounting in short-term priming: 1. Effects of prime duration and prime recognition. *Mem. Cogn.* 30 745–757. 10.3758/BF03196430 12219891

[B13] Kounios J. Kotz S. A. Holcomb P. J. (2000). On the locus of the semantic satiation effect: evidence from event-related brain potentials. *Mem. Cogn.* 28 1366–1377. 10.3758/BF03211837 11219964

[B14] Kuhl B. A. Anderson M. C. (2011). More is not always better: paradoxical effects of repetition on semantic accessibility. *Psychon. Bull. Rev.* 18 964–972. 10.3758/s13423-011-0110-0 21584852

[B15] Lambert W. E. Jakobovits L. A. (1960). Verbal satiation and changes in the intensity of meaning. *J. Exp. Psychol.* 60 376–383. 10.1037/h0045624 13758466

[B16] Lewis M. B. Ellis H. D. (2000). Satiation in name and face recognition. *Mem. Cogn.* 28 783–788. 10.3758/BF03198413 10983452

[B17] Pilotti M. Antrobus J. S. Duff M. (1997). The effect of presemantic acoustic adaptation on semantic “satiation.”. *Mem. Cogn.* 25 305–312. 10.3758/BF03211286 9184482

[B18] Pynte J. (1991). The locus of semantic satiation in category membership decision and acceptability judgment. *J. Psychol. Res.* 20 315–335. 10.1007/BF01074284

[B19] Severance E. Washburn M. F. (1907). The loss of associative power in words after long fixation. *Am. J. Psychol.* 18 182–186. 10.2307/1412411

[B20] Shinkareva S. V. Malave V. L. Mason R. A. Mitchell T. M. Just M. A. (2011). Commonality of neural representations of words and pictures. *NeuroImage* 54 2418–2425. 10.1016/j.neuroimage.2010.10.042 20974270

[B21] Smith L. Klein R. (1990). Evidence for semantic satiation: repeating a category slows subsequent semantic processing. *J. Exp. Psychol.* 16 852–861. 10.1037//0278-7393.16.5.852

[B22] Tan L. H. Liu H.-L. Perfetti C. A. Spinks J. A. Fox P. T. Gao J.-H. (2001). The neural system underlying chinese logograph reading. *NeuroImage* 13 836–846. 10.1006/nimg.2001.0749 11304080

[B23] Tan L. H. Spinks J. A. Eden G. F. Perfetti C. A. Siok W. T. (2005). Reading depends on writing, in Chinese. *Proc. Nat. Acad. Sci. U.S.A.* 102 8781–8785. 10.1073/pnas.0503523102 PMC1150863 15939871

[B24] Tian X. Huber D. E. (2010). Testing an associative account of semantic satiation. *Cogn. Psychol.* 60 267–290. 10.1016/j.cogpsych.2010.01.003 20156620 PMC2882703

[B25] Yuan J. Carr S. Ding G. Fu S. Zhang J. X. (2017). An associative account of orthographic satiation in chinese characters. *Read. Writ.* 30 631–651. 10.1007/s11145-016-9693-1

