# Supplementary material for: A Lexical Representational Mechanism Underlying Verbal Satiation: An Empirical Study With Rarely Used Chinese Characters
Source: Front Psychol. 2019 Oct 2;10:2236. doi: 10.3389/fpsyg.2019.02236 (PMC6783687; doi:10.3389/fpsyg.2019.02236)
Supplement: Supplementary file 1 [file Data_Sheet_1.docx]

Supplementary Material

**Appendix Table A1** Rarely-used Chinese characters and their corresponding matching and mismatching commonly-used characters.

| Rarely-used characters | Matching characters | Mismatching characters |
| --- | --- | --- |
| 殶 | 主 | 业 |
|  | 几 | 刁 |
|  | 又 | 丁 |
| 熼 | 火 | 反 |
|  | 田 | 甘 |
|  | 共 | 衣 |
| 瑉 | 王 | 支 |
|  | 民 | 示 |
|  | 日 | 叉 |
| 蹹 | 足 | 采 |
|  | 合 | 耳 |
|  | 羽 | 羊 |
| 塄 | 土 | 寸 |
|  | 四 | 半 |
|  | 方 | 夫 |
| 峠 | 山 | 凡 |
|  | 上 | 工 |
|  | 下 | 个 |
| 艓 | 舟 | 色 |
|  | 世 | 本 |
|  | 木 | 公 |
| 媆 | 女 | 丸 |
|  | 而 | 舌 |
|  | 大 | 于 |
| 牎 | 片 | 文 |
|  | 匆 | 古 |
|  | 心 | 丰 |
| 砕 | 石 | 乎 |
|  | 九 | 儿 |
|  | 十 | 力 |
| 膃 | 月 | 丹 |
|  | 囚 | 且 |
|  | 皿 | 令 |
